# Berberine attenuates XRCC1‐mediated base excision repair and sensitizes breast cancer cells to the chemotherapeutic drugs

**DOI:** 10.1111/jcmm.14560

**Published:** 2019-07-23

**Authors:** Xingjie Gao, Jing Wang, Meiqi Li, Jia Wang, Jian Lv, Lu Zhang, Caifeng Sun, Jiamei Ji, Wenbo Yang, Zinan Zhao, Weifeng Mao

**Affiliations:** ^1^ Department of Biotechnology, College of Basic Medical Sciences Dalian Medical University Dalian China

**Keywords:** base excision repair, Berberine, breast cancer, DNA damage agents, XRCC1

## Abstract

Berberine (BBR) is a natural isoquinoline alkaloid, which is used in traditional medicine for its anti‐microbial, anti‐protozoal, anti‐diarrhoeal activities. Berberine interacts with DNA and displays anti‐cancer activities, yet its effects on cellular DNA repair and on synthetic treatments with chemotherapeutic drugs remain unclear. In this study, we investigated the effects of BBR on DNA repair and on sensitization of breast cancer cells to different types of DNA damage anti‐tumoural drugs. We found BBR arrested cells in the cell cycle S phase and induced DNA breaks. Cell growth analysis showed BBR sensitized MDA‐MB‐231 cells to cisplatin, camptothecin and methyl methanesulfonate; however, BBR had no synergistic effects with hydroxurea and olaparib. These results suggest BBR only affects specific DNA repair pathways. Western blot showed BBR down‐regulated XRCC1 expressions, and the rescued XRCC1 recovered the resistance of cancer cells to BBR. Therefore, we conclude that BBR interferes with XRCC1‐mediated base excision repair to sensitize cancer cells to chemotherapeutic drugs. These finding can contribute to understanding the effects of BBR on cellular DNA repair and the clinical employment of BBR in treatment of breast cancer.

## 1 INTRODUCTION

Breast cancer is the most common malignant tumours among women, with approximately 458 000 deaths each year across the globe.^1^, ^2^ Multiple strategies are employed to treat breast cancer, which include surgery, radiotherapy, chemotherapy and hormone therapy.^3^, ^4^, ^5^ Among the various therapy strategies, DNA damage agents are wildly used in breast cancer treatment, especially to triple‐negative breast cancer (TNBC). TNBC lacks of oestrogen receptor, progesterone receptor and epidermal growth receptor 2, thus is insensitive to anti‐hormone receptor‐targeted drugs, and the chemotherapy is the current efficient method for TNBC treatment.^6^, ^7^, ^8^, ^9^ A range of chemotherapeutic drugs are clinically used for TNBC treatments, in which cisplatin is one of the current most effective one.^10^ While the efficacies of the chemotherapeutic drugs are often low due to the drug resistance and side effects, which has become a major challenge in the successful treatment of breast cancer, especially in TNBC.^11^, ^12^, ^13^

Berberine (BBR) is an isoquinoline alkaloid extracted from the rhizomes of Coptis, which displays multiple biochemical functions and anti‐microbial and anti‐inflammatory activities.^14^, ^15^ BBR also exhibits anti‐cancer activities through suppressing cell proliferation and inducing tumour cell apoptosis.^16^, ^17^, ^18^, ^19^, ^20^, ^21^, ^22^ BBR reportedly directly binds with DNA, radio‐sensitizes lung and oesophageal cancer cells and sensitizes ovarian cancer cells to cisplatin and PARP inhibitors.^23^, ^24^ Consequently, BBR may interfere with cellular DNA repair and would be a promising adjuvant agent to sensitize breast cancer cells to chemotherapeutic drugs. However, the influence of BBR on DNA repair was not well defined.

In this study, we attempted to analyse the DNA repair pathways influenced by BBR. We analysed the effect of BBR on cell cycle and on cellular DNA breaks. We also tested the cell proliferation upon stimulating with BBR in combination with DNA damage agents including cisplatin, camptothecin, methyl methanesulfonate (MMS), hydroxurea (HU) and olaparib. Cisplatin is classified as an alkylating agent and is the most widely used anti‐tumour drug, which reacts with DNA to form interstrand crosslinks to inhibit its replication.^25^, ^26^ MMS is also an alkylating agent to methylate DNA on N7‐G and N3‐A, which would cause single‐strand breaks (SSB) and the repair is dependent on XRCC1‐mediated base excision repair (BER).^27^ Camptothecin is a topoisomerase inhibitor that prevents DNA ligations and generates DNA breaks at the sites of DNA replication.^28^ HU is an anti‐tumoural drug classified as an antimetabolite one, which inhibits ribonucleotide reductase to block the formation of deoxyribonucleotides.^29^ Olaparib is a poly‐ADP‐ribose polymerase inhibitor, which prevents the parylation of DNA repair factors and is a promising anti‐tumour drug in breast cancer treatment.^30^ After finding the potential DNA repair pathway affected by BBR, we analysed the relevant DNA repair factors in BBR‐treated cancer cells to find out the factors that were affected by BBR. These studies will contribute to understanding the acts of BBR in cellular DNA repair, also the clinical employments of BBR in chemotherapeutic and radiotherapeutic treatment of breast cancer.

## 2 MATERIALS

### 2.1 Cell culture and transfection

Human breast cancer cell lines, BT549 and MDA‐MB‐231, were achieved from American Tissue Culture Colection (ATCC). Cells were cultured in RPMI 1640 (Hyclone) and DMEM (Gibco) medium with 10% FBS (Biological Industries, Kibbutz Beit Haemek), 1% penicillin and streptomycin (Gibico). Cells were transfected by Lipofectamine 2000 according to the manufacturer's instructions (Invitrogen).

### 2.2 Reagents and antibodies

BBR was obtained from Sigma (Sigma‐Aldrich). HRP‐conjugated antibodies and antibodies to PARP1 (13371‐1), Rad51 (14961‐1‐AP), ERCC1 (1456‐1), XRCC1 (21468‐1‐AP), β‐actin (66009‐1) were from Proteintech. Antibody to poly‐ADP‐ribose (ab14460) is from Abcam. Alexa fluor 488‐conjugated secondary antibody was purchased from Proteintech. Hydroxyurea, MMS and olaparib were purchased from MedChemExpress. Cisplatin and camptothecin were purchased from Selleck. pCMV‐His (CV003, Sino Biological), pCMV‐His‐hXRCC1s (HG15275‐NH, Sino Biological), pCMV‐His‐hPARP1 (HG11040‐NH, Sino Biological) vectors were purchased from Sino Biological.

### 2.3 MTT assay

Cell viability was measured by MTT assay. 6 × 10^3^ cells were seeded in a well in 96‐well culture plates and cultured overnight. Cells were treated with BBR for 24 hours. MTT (Solabio) was added into each well; after 4 hours, the cells were measured at 492 nm using a microplate photometer (Thermo Fisher Scientific).

### 2.4 Cell cycle analysis

Approximately 2 × 10^6^ cells were exposed to BBR. 24 hours after treatment, the cells were harvested, washed and centrifuged at 400 *g* for 5 minutes. The cells were resuspended in 70% ethanol at 4°C overnight, washed and spun down. The cells were resuspended in 200 μL of RNAase A (1 mg/mL) and propidium iodide solution with a final concentration of 50 μg/mL (Sangon Inc). The fluorescence of cells was analysed through flow cytometry, and the results were analysed by Flow Plus software.

### 2.5 Comet assay

Cells were treated with different doses of BBR for one night. 3 × 10^4^ cells were isolated for analysis. Dipped 1% normal melting agarose into a frosted microscope slide, 10 μL cells were mixed with 90 μL 0.7% low melting agarose. The microscope slide was treated with ice‐cold lysis buffer (100 mmol/L EDTA, 2.5 mol/L NaCl, 10 mmol/L Tris Base, 100 mmol/L EDTA, 1% sodium sarcosinate, 10% DMSO and 1% Triton X‐100) for 2 hours. Then the cells were denatured in alkaline buffer (10 mmol/L EDTA, pH 13) for 40 minutes and electrophoresed for 30 minutes at 25 V. The slides were washed three times by 0.4 mol/L Tris‐HCl (pH7.5), stained by 5 μg/mL ethidium bromide for 10 minutes. 30 cells were analysed in each slide using a fluorescence microscope.

### 2.6 Reverse transcription‐polymerase chain reaction assay

Cells were cultured in 3 cm plate and treated with different doses of BBR for 24 hours. mRNA was extracted by Trizol. Primers, XRCC1F: 5′ TGGACATTGGGAATGATGGC 3′; XRCC1R: 5′ CTCGGAAGGGGACATGAAAG 3′; β‐actinF: 5′ GATTCCTATGTGGGCGACG 3′ β‐actinR: 5′ TGTGGTGCCAGATTTTCTCC 3′, were used to amplify XRCC1 and actin genes. 200 ng template mRNA and 0.4 μmol/L primers were used in 25 μL Reverse transcription‐polymerase chain reaction (RT‐PCR) system through PrimeScript™ One Step RT‐PCR Kit Ver.2 (RR055A, Takara). The production of PCR was electrophoresed in 2% agarose gel.

### 2.7 Western blot

Proteins were extracted using ice‐cold RIPA buffer with 1 × protease inhibitors (Selleck) and 1 mmol/L PMSF (Sangon Inc). Lysates were separated by 12% SDS‐PAGE, transferred to 0.22 μm PVDF membranes. Various antibodies (1:500) were used to detect proteins.

### 2.8 Drug combination index analysis

The combination index (CI) was calculated through the software CompuSyn (Biosoft). CI <0.7 was considered as synergism. CI <0.5 was considered as strong synergism. The IC50 values of BBR with different doses of drugs were calculated by Graphpad Prism 5 (Graphpad).

### 2.9 Statistical analysis

Data analysis was completed by GraphPad Prism 6.0 software. *t* Test was used to determine the significance; *P < .05, **P < .01, ***P < .001, were considered statistically significant. All experiments were performed in triplicate. Data are expressed as the mean ± SD.

## 3 RESULTS

### 3.1 Berberine suppresses the growth of triple‐negative breast cancer cell, MDA‐MB‐231, and arrests cells on S phase of cell cycle

**Figure 1:**
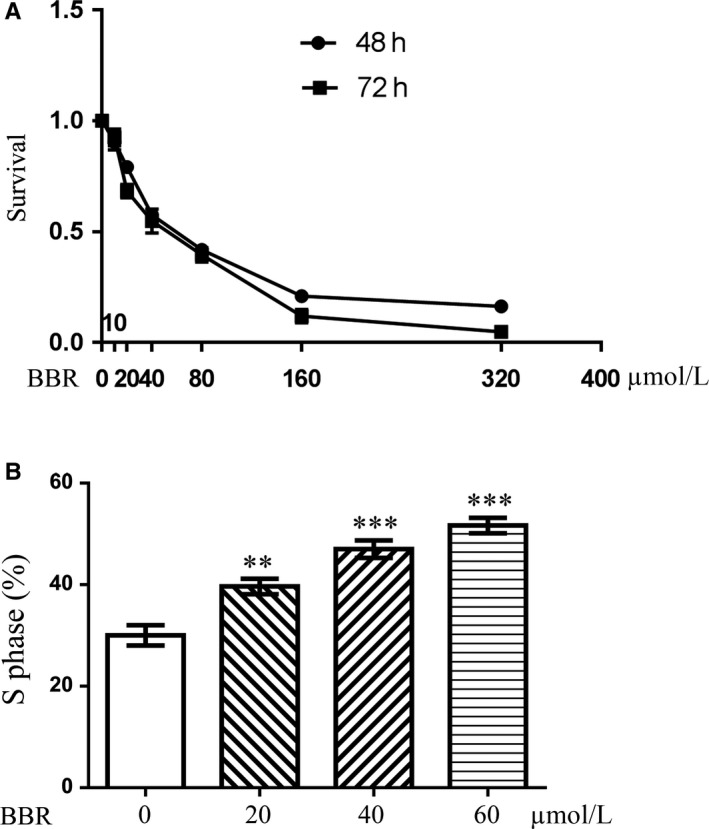
Berberine inhibits the growth of MDA‐MB‐231 breast cancer cells and arrests cells in S phase of cell cycles. After 48 and 72 h treatment, the cell viability was measured by MTT assay. BBR suppressed the growth of MDA‐MB‐231 cells in a dose‐dependent manner (A). The cell cycle was detected through propidium iodide staining and FACS analysis, which showed BBR (20, 40 60 μmol/L) arrested MDA‐MB‐231 cells in S phase of cell cycles (B). The data are presented as mean ± SD of three tests. *t* Test, ***P < .001 as compared with the control

We analysed the effects of berberine on the growth of MDA‐MB‐231 cells through MTT assay. After 24 hours treatment, BBR suppresses the proliferation of MDA‐MB‐231 cells (Figure 1A). The cell cycle analysis showed BBR arrest the cells on S phase, which suggests BBR may affect DNA replication or delay DNA repair upon the damages (Figure 1B).

### 3.2 Berberine induces DNA breaks

**Figure 2:**
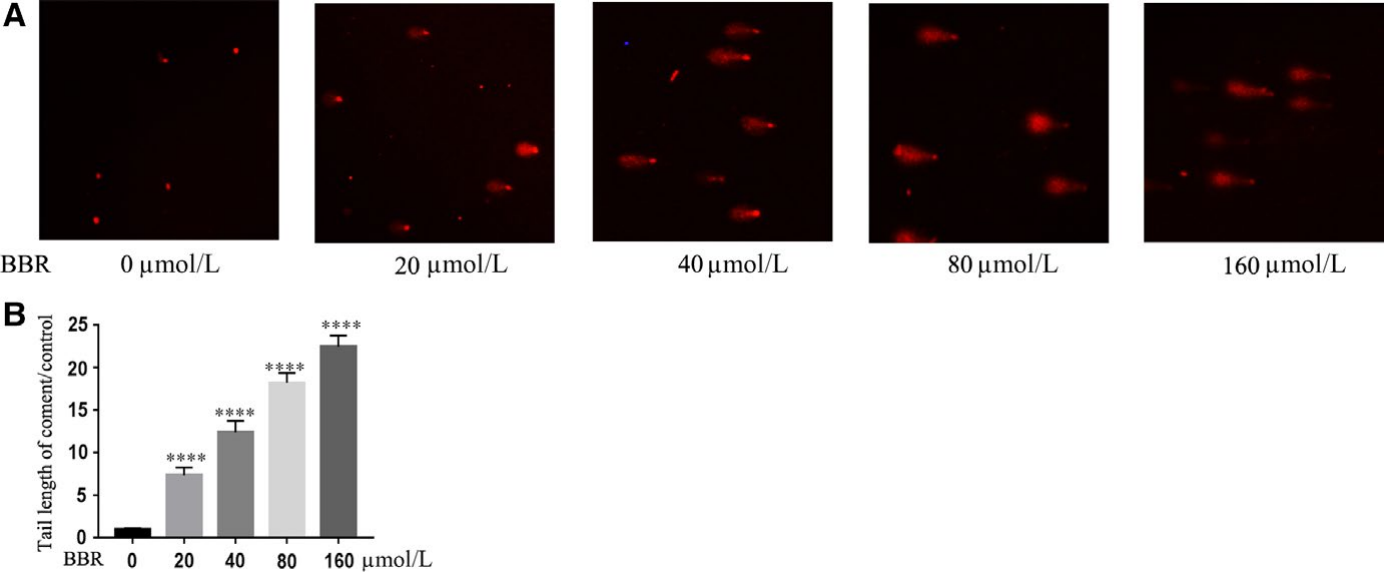
Berberine induces DNA breaks. Comet assay showed 20, 40, 80 and 160 μmol/L BBR promoted the tails of comet in the comet assay (A). The folds of changes of comet tails upon different doses of BBR were quantified (control was set as 1) (B). 30 tails were analysed in this assay. *t* Test, ***P < .001 as compared with the control

We next analysed the effects of BBR on cellular DNA damages. Through comet assay, we found BBR increased the lengths of comet tails in a dose‐dependent manner, which demonstrates BBR induces DNA breaks. At the treatment of 40 µmol/L dose of BBR, the tails of comet were increased, while 160 µmol/L BBR increased much more folds of tails of comet compared with the control (Figure 2A, B). These results demonstrate that BBR induces cellular DNA damage. As BBR also arrests cell cycle in S phase, it is possible that BBR interferes with the cellular DNA repair.

### 3.3 Berberine sensitizes MDA‐MB‐231 cells to cisplatin, camptothecin, MMS, but not to HU and olaparib

**Figure 3:**
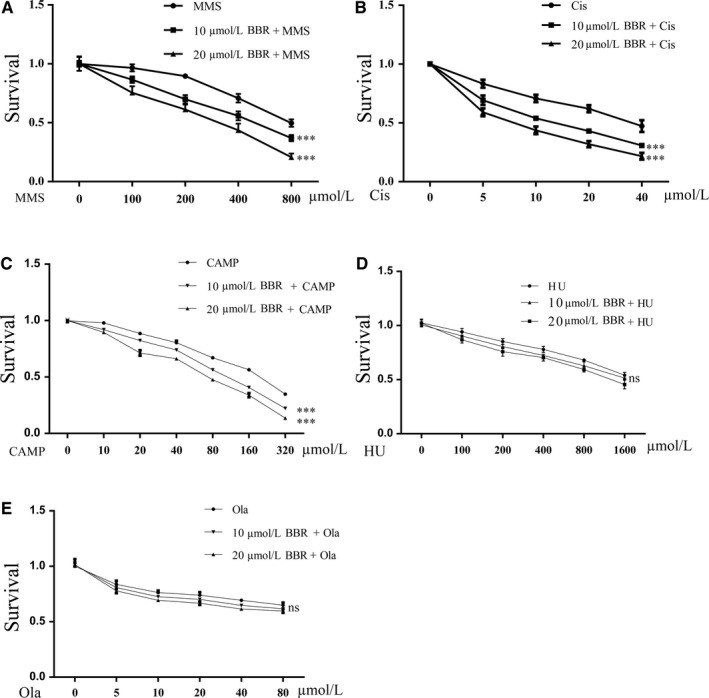
Berberine sensitizes breast cancer cells to cisplatin, camptothecin and MMS, but has no synergistic effects with HU and olaparib. MTT assay was used to analyse the viability of MDA‐MB‐231 cell treated with BBR and DNA damage anti‐tumoural drugs including cisplatin (Cis), camptothecin (CAMP), methyl methanesulfonate (MMS), hydroxyurea (HU) and olaparib (Ola; A‐E). 10 μmol/L BBR sensitizes breast cancer cells to cisplatin, camptothecin and MMS, but has no synergistic effects with HU and olaparib. *t* Test, ns, no significant difference, **P < .01, ***P < .001 as compared with the control

Different types of DNA damage drugs were used to check the potential DNA repair pathway affected by BBR. After 24 hours treatment, BBR increased the sensitization of TNBC to cisplatin, camptothecin, MMS, but had no effects on hydroxyurea (HU) and olaparib (Figure 3A‐E). The combination index (CI) of synergistic effects between BBR and different drugs were analysed through CompuSyn. 20 μmol/L BBR has synergistic effects (CI < 0.7) with MMS and has strong synergistic effects (CI < 0.5) with cisplatin and camptothecin. However, BBR has no synergistic effects with HU and olaparib (Table 1, Figure S1).

**Table 1:** The combination index (CI) of BBR with HU, CIS, MMS and Ola

BBR (μmol/L)	CAMP (μmol/L)	CI^a^	CIS(μmol/L)	CI^a^	HU (μmol/L)	CI	MMS (μmol/L)	CI^a^	Ola (μmol/L)	CI
10.0	10.0	0.656	5.0	0.488	100.0	1.00	100.0	0.606	5.0	0.823
10.0	20.0	0.517	10.0	0.376	200.0	0.89	200.0	0.410	10.0	0.918
10.0	40.0	0.455	20.0	0.430	400.0	0.99	400.0	0.448	20.0	0.952
10.0	80.0	0.420	40.0	0.434	800.0	0.81	800.0	0.573	40.0	0.928
10.0	160.0	0.396			1600.0	0.89			80.0	0.856
10.0	320.0	0.376								

BBR (μmol/L)	CAMP (μmol/L)	CI^b^	CIS (μmol/L)	CI^b^	HU (μmol/L)	CI	MMS (μmol/L)	CI^a^	Ola (μmol/L)	CI
20.0	10.0	0.692	5.0	0.331	100.0	0.915	100.0	0.443	5.0	0.677
20.0	20.0	0.423	10.0	0.248	200.0	0.808	200.0	0.373	10.0	0.573
20.0	40.0	0.385	20.0	0.249	400.0	0.873	400.0	0.350	20.0	0.657
20.0	80.0	0.262	40.0	0.229	800.0	0.783	800.0	0.303	40.0	0.715
20.0	160.0	0.263			1600.0	0.837			80.0	0.733
20.0	320.0	0.168								

a Synergism.

b Strong synergism.

At the dose of 10 and 20 μmol/L, BBR effectively increased the sensitization of TNBC to cisplatin, camptothecin and MMS. Cisplatin is considered as a type of alkylating agent and one of the most effective drugs used to treat cancers.^11^, ^31^ BER and HR reportedly repair the DNA damages induced by cisplatin.^32^ MMS is also an alkylating agent and induces single‐strand breaks, which is repaired by XRCC1‐dependent BER.^33^, ^34^ Camptothecin is a topoisomerase I inhibitor acting to induce DNA breaks. The cancer cells over‐expressed XRCC1 resist to camptothecin and the XRCC1 deficient cells are sensitive to camptothecin.^35^, ^36^ Therefore, these data suggest XRCC1 would be an important target for BBR to function in DNA repair. We also tested the synergistic effects between BBR and HU, as well as between BBR and olaparib. Olaparib is a PARP1 inhibitor and HU is to inhibit ribonucleotide reductase. We found 10 μM BBR cannot increase the sensitization of TNBC to olaparib and HU, which suggest BBR may not affect the repair pathways in which olaparib and HU are involved.

### 3.4 Berberine severely down‐regulates the levels XRCC1 and ERCC1, slightly decreases RAD51，but has no effects on PARP1

**Figure 4:**
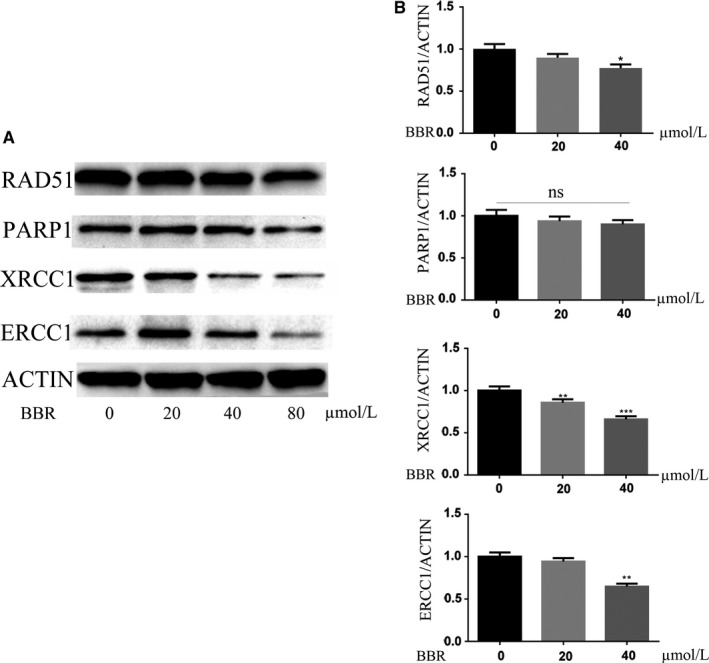
Western analysis of cellular RAD51, XRCC1, PARP1 and ECRR1 expressions after treatment with BBR. Western results showed BBR obviously decreased the expressions of XRCC1 and ERCC1, slightly decreased RAD51, but had no effects on PARP1 (A). The western assays were repeated for three times, and the results were quantified (B). *t* Test, **P < .01, ***P < .001 as compared with the control

We next analysed the effects of low doses of BBR on expressions of DNA repair factors through western blot, which showed BBR slightly decreased the expressions of Rad51, but severely decreased XRCC1 and ERCC1 in a dose‐dependent manner (Figure 4A, 4). Rad51 plays vital roles in HR, thus results suggested BBR may slightly affect the HR efficiency through regulating Rad51 expression. In BER, XRCC1 is a scaffolding protein to recruit Ligase III, DNA polymerase and PARP1 to repair the single‐strand breaks. Therefore, BBR may impair the BER efficacy through down‐regulating XRCC1. ERCC1 is a key factor in nuclear excision repair (NER) and has critical roles in removing the DNA adducts caused by cisplatin. PARP1 is a member of poly‐ADP‐ribose polymerase (PARP) family, which is to add poly‐ADP‐ribose to DNA repair factors to affect their binding to chromatin, thus it is considered as a DNA break sensor and involved in different DNA repair pathways. We found low doses of BBR could not influence the levels of PARP1. We also detected the cellular free poly‐ADP‐ribose (PAR) in MDA‐MB‐231 cells treated with BBR. Unexpected, BBR increased the cellular free PAR (Figure S2A), which suggest BBR may regulate the enzymatic activity of PARP. However, we find BBR didn't change the levels of PARP1 (Figure 4A, 4). PARP family includes 16 family members, in which PARP1, PARP2, PARP4 and PARP5 have enzymatic activity to add PAR into the target proteins.^37^ Thus, BBR might not directly act on PARP1, but probably on the other PARP family members to regulate the parylation process. We also tested the transcript levels of XRCC1 in cells treated with BBR, while the results showed BBR didn't affect the XRCC1 transcription (Figure S2B). These data showed BBR decreased XRCC1 through the process of translation or post‐translational modification.

### 3.5 Rescue of XRCC1 helps the breast cancer cells to resist BBR, while the over‐expressed PARP1 cannot influence the effects of BBR on the growth of breast cancer cells

**Figure 5:**
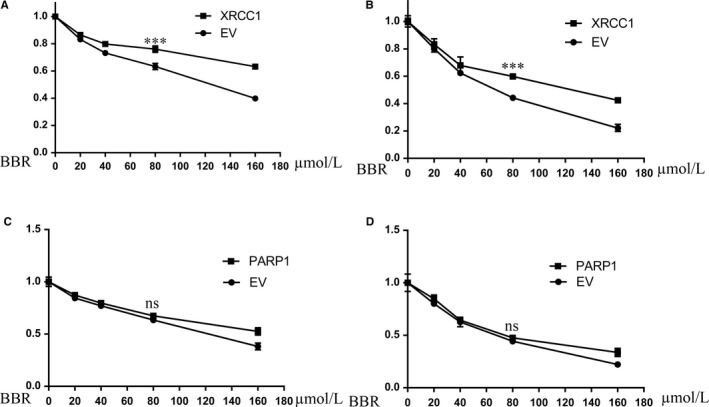
MDA‐MB‐231 and BT549 cells complemented with XRCC1 resist to BBR. MDA‐MB‐231 and BT549 cells over‐expressed PARP1 didn't exhibit resistance to BBR. After treatment with BBR for 24 h, MTT assay shows the MDA‐MB‐231 and BT549 cells complemented with XRCC1 resist to BBR compared with cells transfected with empty vector (A). MTT assay shows the over‐expressed PARP1 did not help cancer cells to resist to BBR compared with cells transfected with empty vector (B). *t* Test, ***P < .001, **P < .01, *P < .05

After finding BBR obviously decreases the levels of XRCC1, we next analyse whether XRCC1 is the vital factor for BBR to affect cellular DNA damage and to influence the cell growth. We artificially expressed XRCC1 in two TNBC, MDA‐MB231 and BT549, and tested the growth of cancer cells treated with BBR (Figure S3A). We found the rescued XRCC1 recovered the resistance of cancer cells to BBR (Figure 5A, 5). These data demonstrate XRCC1 is an important target for BBR to affect cellular DNA repair and cell growth. As we found BBR didn't change PARP1 expressions but affect the cellular free PAR, we over‐expressed PARP1 in cancer cells to detect the cell growth after treatment with BBR (Figure 5C, 5; Figure S3B). The over‐expressed PARP1 did not help the cells to resist to 80 μmol/L dose of BBR. These results demonstrate that PARP1 is not involved in the effects of BBR on DNA repair and cell growth.

## 4 DISCUSSION

BBR has anti‐microbial, and‐protozoal, anti‐diarrhoeal activities, thus it is widely used in treatments of metabolic and neurological problems in traditional medicine. More recent papers demonstrate that BBR suppresses the growth of various types of tumours through activating apoptosis. BBR reported directly interacts with DNA to form a complex, while the roles of BBR in DNA repair are still not well defined. In this study, we found BBR induced DNA damage and sensitizes cancer cells to DNA damage agents, cisplatin, MMS and camptothecin, which suggests BBR interferes with cellular DNA repair. Various sorts of chemotherapeutic drugs cause different DNA damages that are repaired by the relevant pathways. The DNA interstrands caused by cisplatin can be repaired by BER and nucleotide excision repair. The DNA damages induced by MMS were well known to be repaired by XRCC1‐mediated BER. XRCC1 also reportedly is vital to repair the DNA damages caused by camptothecin. Therefore, XRCC1 would be the potential target for BBR to act to influence the cellular DNA repair. Analysis of the effects of BBR on the levels of DNA repair factors, we found BBR clearly decreases XRCC1 and slightly decreases Rad51, while the levels of PARP1 were not changed by the same dose of BBR. The rescued XRCC1 help TNBC to resist to BBR. Their results demonstrate XRCC1 is a vital factor for BBR function in DNA repair and sensitization of cancer cells to anti‐tumoural drugs. We also found BBR decreased the levels of ERCC1, as ERCC1 is a vital factor in NER to repair the DNA damage caused by cisplatin, BBR may function in interfering with NER to sensitize cancer cells to chemotherapeutic drugs.

We analysed the synergistic effects of BBR with HU in treating TNBC, but didn't find the sensitization caused by BBR. HU is an anti‐tumoural drugs classified as an antimetabolite one, which inhibits ribonucleoside diphosphate reductase, therefore causing deoxyribonucleotide depletion, preventing cells from leaving the cell cycle G1/S phase and resulting in DNA breaks near replication forks. However, our data showed there is no obviously synergistic effect between BBR and HU, which suggests BBR would interfere with DNA repair, rather than directly induces DNA breaks.

Currently, PARP1 inhibitors become promising drugs in breast cancer treatment, which can delay the parylation of DNA repair factors, such as Ku70, DNA‐PKcs, BARD1 etc.^38^, ^39^ Thus, inhibition of PARP1 would affect the interactions between DNA repair factors and the DNA breaks to impair the DNA damage response. In this study, at the dose of 10 and 20 μmol/L, BBR did not increase the sensitization of breast cancer cells to olaparib. We also found 10 and 20 μmol/L BBR did not change the expression levels of PARP1. These results suggest BBR is not directly involved in PARP1‐mediated DNA damage response. However, we found BBR induced more cellular free PAR, which suggest BBR influences the cellular parylation process although BBR might not directly function in the activity of PARP1.

In sum, we studied the roles of BBR in DNA repair and in synthetic sensitization with different DNA damage anti‐tumoural drugs. We found BBR interferes with cellular DNA repair, rather than directly inducing DNA breaks. BBR impaired BER through down‐regulating XRCC1, and BBR may also affect HR through regulating Rad51. HR and BER are two important DNA repair pathways in tumour chemotherapeutic resistance. Therefore, these results demonstrate BBR attenuates XRCC1‐mediated BER and can be employed to sensitize TNBC to the relevant chemotherapeutic DNA damage agents.

## CONFLICT OF INTEREST

The authors have no ethical conflicts to disclose.

## AUTHOR CONTRIBUTIONS

Xingjie Gao, Jing Wang and Meiqi Li performed the experiments and collected the data. Jia Wang, Jian Lv and Lu Zhang also performed some parts of the assays. Caifeng Sun, Jiamei Ji, Wenbo Yang and Zinan Zhao collected data and performed statistical analysis. Weifeng Mao searched literature, designed this study and wrote the manuscript.

## Data Availability

I confirm that my article contains a Data Availability Statement. I confirm that I have included a citation for available data in my references section.
