# Supplementary figures and images for: Berberine attenuates XRCC1‐mediated base excision repair and sensitizes breast cancer cells to the chemotherapeutic drugs
Source: J Cell Mol Med. 2019 Jul 23;23(10):6797–804. doi: 10.1111/jcmm.14560 (PMC6787507; doi:10.1111/jcmm.14560)

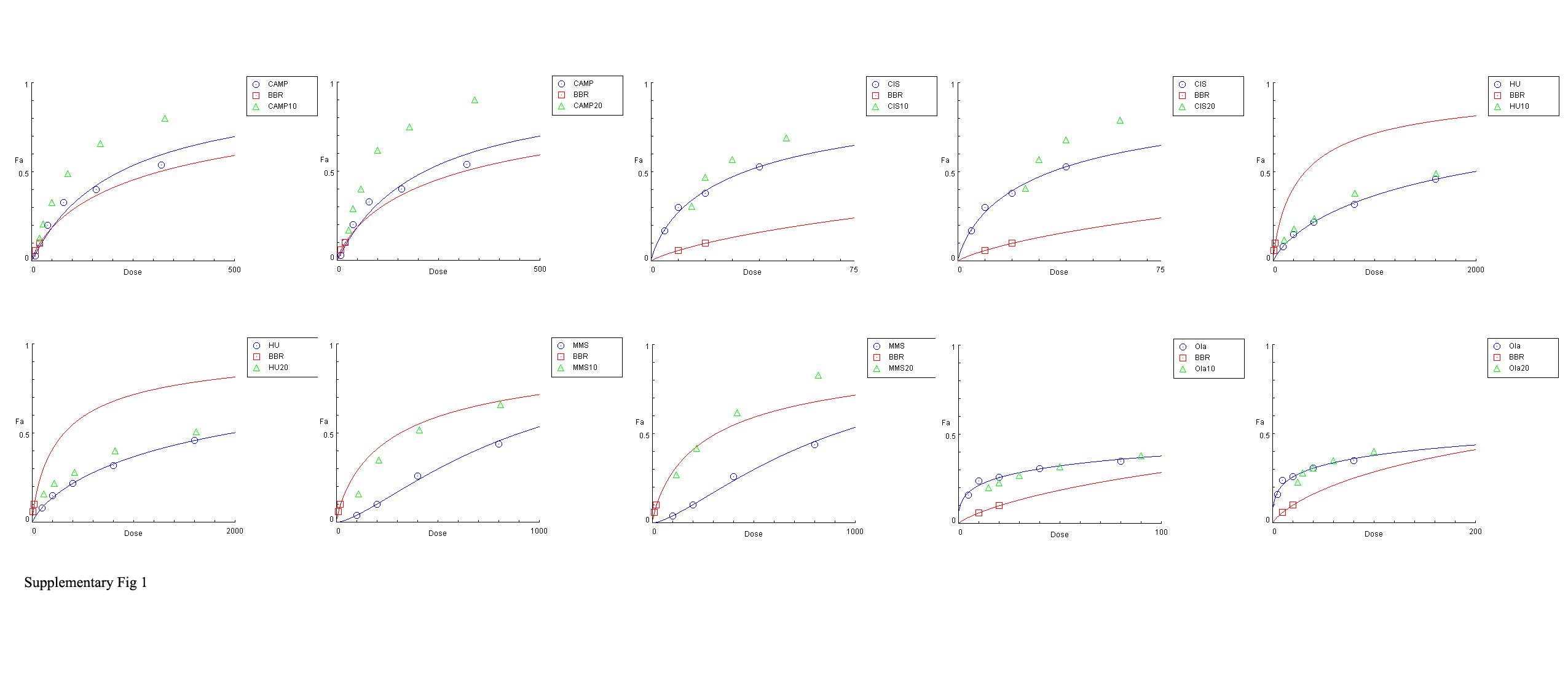

Supplement: Supplementary file 1 [file JCMM-23-6797-s001.tif]

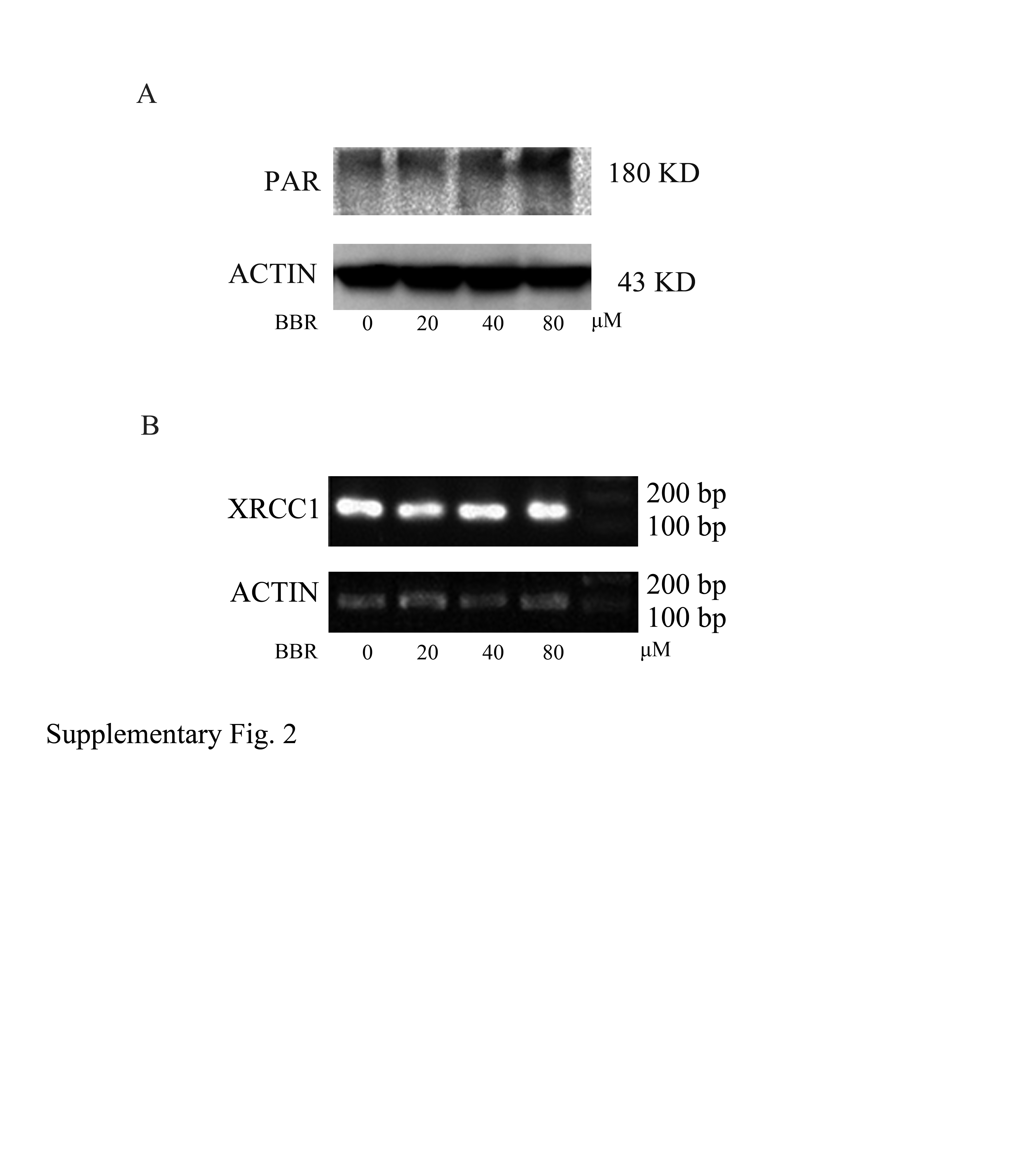

Supplement: Supplementary file 2 [file JCMM-23-6797-s002.tif]

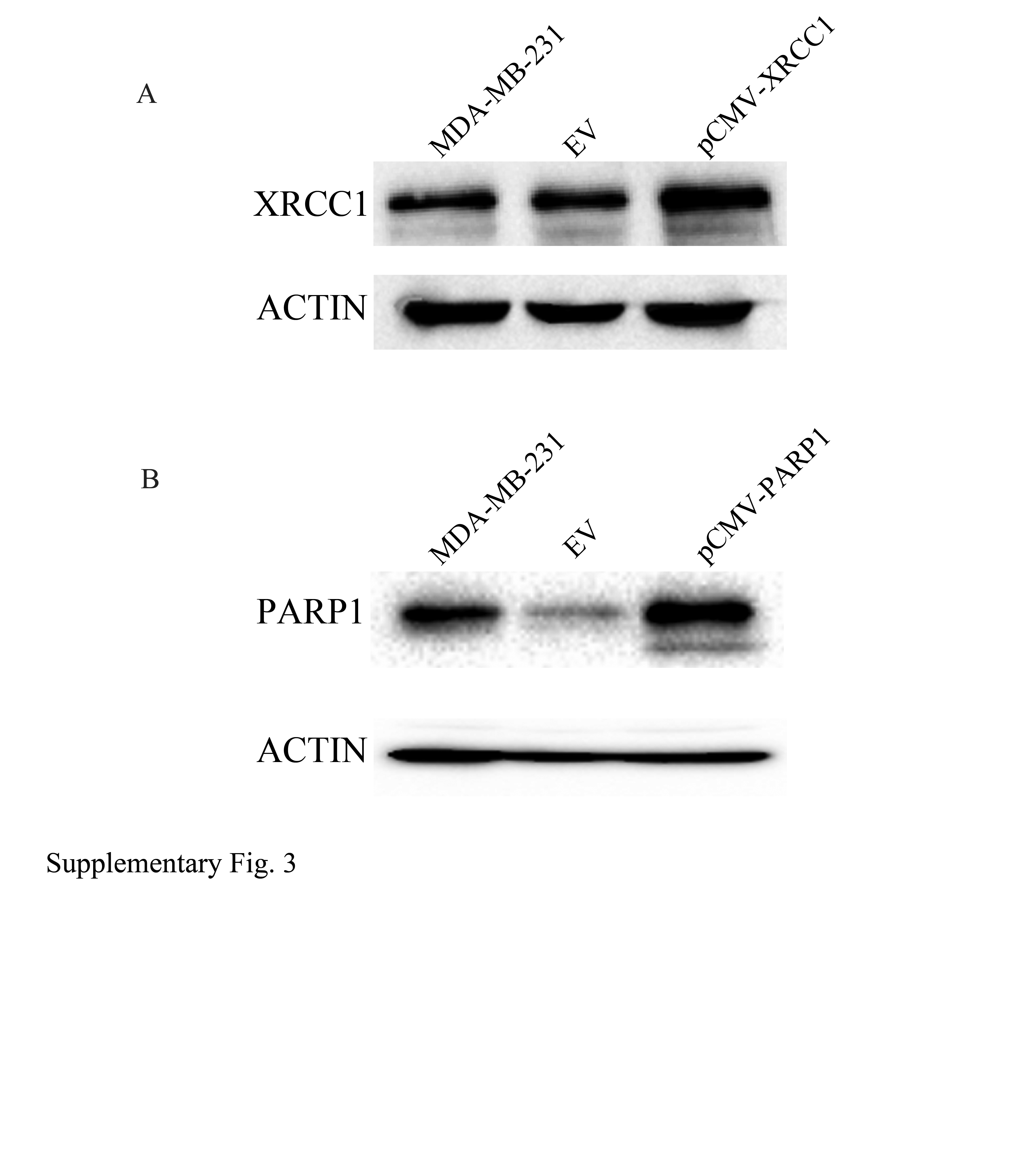

Supplement: Supplementary file 3 [file JCMM-23-6797-s003.tif]
